# Supplementary material for: PI3K/Akt1 signalling specifies foregut precursors by generating regionalized extra-cellular matrix
Source: eLife. 2013 Dec 24;2:e00806. doi: 10.7554/eLife.00806 (PMC3871052; doi:10.7554/eLife.00806)
Supplement: Supplementary file 1. — Inhibitor doses and cell survival. DOI: http://dx.doi.org/10.7554/eLife.00806.025 [file elife00806s004.pdf]

A

	PD03	SP600125	SB239063	LY249002*
High (H)	3 mM	10 mM	100 mM	40 mM
Medium (M)	-	5 mM	50 mM	20 mM
Low (L)	1 mM	1 mM	10 mM	10 mM

*Doses of LY used in this work are: Low from d3.5 to d4.5, and Medium from d4.5 onwards

B

Cell Survival	PD03			SP600125			SB239063			LY249002		
	H	M	L	H	M	L	H	M	L	H	M	L
Phase1	++	ND	++	-	-	-	+++	+++	++	-	-	-
Phase 2	++	ND	++	-	-	+	+++	+++	++	+	++	++

-, no cell survival
+, cell death significantly increased
++ , cell death similar to control cultures
+++, cell death significantly reduced
ND, no data

Supplementary file 1.
Inhibitor doses and cell survival
(A) Experimental dose of the inhibitors used during phases 1 and 2 of differentiation and, **(B)** Cell survival in response to inhibitor treatment during ESC differentiation.
